# Access to inpatient mood management services after stroke in Australian acute and rehabilitation hospitals

**DOI:** 10.1177/02692155241232990

**Published:** 2024-02-22

**Authors:** Shaun L Hancock, Tara Purvis, Tharshanah Thayabaranathan, Rene Stolwyk, Jan Cameron, Lachlan L Dalli, Megan Reyneke, Monique F Kilkenny, Kelvin Hill, Dominique A Cadilhac

**Affiliations:** 1 Stroke and Ageing Research Group, Department of Medicine, School of Clinical Sciences at Monash Health, 2541 Monash University, Clayton, VIC, Australia; 2 School of Psychological Sciences, Turner Institute for Brain and Mental Health, Faculty of Medicine, Nursing and Health Sciences, 2541 Monash University, Clayton, VIC, Australia; 3 Florey Institute of Neuroscience and Mental Health, Heidelberg, VIC, Australia; 4 Stroke Foundation, Melbourne, VIC, Australia

**Keywords:** Stroke, health care, health psychology, depression, quality improvement

## Abstract

**Objective:**

Post-stroke mental health impairments are common, but under-assessed and under-treated. We aim to describe trends in the provision of mood management to patients with stroke, and describe factors associated with adoption of national mood management recommendations for stroke within Australian hospitals.

**Design:**

Secondary analysis of cross-sectional data from the biennial Stroke Foundation Audit Program.

**Setting:**

Participating acute (2011–2021) and rehabilitation hospitals (2012–2020) in Australia.

**Participants:**

In the acute audit, 22,937 stroke cases were included from 133 hospitals. In the rehabilitation audit, 15,891 stroke cases were included from 127 hospitals.

**Main measures:**

Hospital- and patient-level mood management processes.

**Results:**

Among 133 acute hospitals (22,937 stroke episodes), improvements were made between 2011 and 2021 in utilization of mood screening (17% [2011], 33% [2021]; *p *< 0.001) and access to psychologists during hospital stay (18% [2011], 45% [2021]; *p *< 0.001). There was no change in access to a psychologist among those with a mood impairment (*p *= 0.34). Among 127 rehabilitation hospitals (15,891 stroke episodes) improvements were observed for mood screening (35% [2012], 61% [2020]; *p *< 0.001), and access to a psychologist during hospital stay (38% [2012], 68% [2020]; *p *< 0.001) and among those with a mood-impairment (30% [2012], 50% [2020]; *p *< 0.001). Factors associated with receiving mood management processes included: younger age, not requiring an interpreter and longer length of stay.

**Conclusions:**

Adherence to mood management recommendations has improved over 10 years within Australian hospitals. Those aged over 65, requiring an interpreter, or with shorter hospital stays are at risk of missing out on appropriate mood management.

## Introduction

Approximately 41,300 people are admitted to Australian hospitals each year with stroke.^1^ Within 6 months post-stroke, one in three survivors experience a mental health impairment,^2^ the most common being depression, anxiety, fatigue and apathy.^3^ Mental health impairments can have a variety of impacts on the survivor of stroke, including reduced participation in rehabilitation therapy and poor recovery.^4^ Timely diagnosis of mental health impairments is essential for facilitating early access to treatment, thereby improving a patient's prognosis^5^ and reducing economic burden.^6^ However, identifying mental health impairments in people with stroke can be difficult due to communication difficulties, cognitive impairments and stigma.^7,8^

The Australian and New Zealand clinical guidelines for stroke management are living guidelines that provide updated recommendations for best-practice care, including the management of mental health impairments.^9^ In the Australian and New Zealand stroke clinical guidelines, “mood” refers to a patient's state of mind or emotion,^10^ and includes conditions such as depression, anxiety and emotional lability.^9^ The current recommendations, introduced in 2017, include a two-step process for assessing suspected mental health impairments in stroke survivors: (a) use of a standardized and validated mood screening scale for use in people with stroke and (b) diagnosis should only be made following clinical interview.^9^ Within these guidelines there is a lack of evidence guiding who should be screened and when.^9^ Previous versions of the clinical guidelines recommend all patients have their mood assessed.^11^ The standardized screening tools do not provide a diagnosis for mental health impairments, and are only used to determine if symptoms are present.^12^ There are currently no guidelines in Australia and New Zealand on the most appropriate management approach (e.g. referral to a psychiatrist or psychologist) for survivors of stroke who are identified as having a mood impairment.

Despite the high prevalence and consequences for recovery, mental health impairments are often under-assessed, under-diagnosed and under-treated.^8^ Early detection and proper management of patients prior to hospital discharge is critical, particularly as most survivors of stroke report that their mental health needs are not met at 90–180 days post-event.^13^ Therefore, the aims of the current study are to investigate mood screening and management in acute and rehabilitation hospitals in Australia to determine (a) whether utilization has changed over time and (b) factors associated with the adoption of mood management recommendations. We hypothesize that utilization of mood management processes has improved over the 10-year period.

## Methods

In Australia, stroke care is delivered in free-standing and co-located acute and rehabilitation inpatient hospitals. Mood management and psychological services are provided in both acute and rehabilitation hospitals. Over the last two decades, the Stroke Foundation has conducted a biennial Audit Program of stroke care in participating Australian acute and rehabilitation hospitals in alternating years.^14^ The Acute Services audit covers hospitals providing acute care following a new stroke event, from arrival to discharge (including to the community) or transfer to inpatient rehabilitation.^15^ The Rehabilitation Services audit covers hospitals providing inpatient rehabilitation care for patients with stroke from arrival to discharge (including to the community) or transfer to another inpatient facility.^16^ Both audits are used to assess adherence to stroke clinical guidelines including the provision of mood screening and subsequent management.

### Data sources and study design

The Stroke Foundation Audit Programs (acute and rehabilitation) comprise two components: (a) an organizational survey, which includes data on stroke service characteristics that have been self-reported by a clinical representative from Australian hospitals delivering stroke care (e.g. hospital access to psychologists involved in the management of patients with stroke) and (b) a clinical medical record audit, which includes retrospective patient-level data from approximately 40 consecutive patients from each participating hospital on the processes (e.g. mood screening) received during admission.

De-identified, cross-sectional data were obtained from the acute audit (2011, 2013, 2015, 2017, 2019 and 2021) and the rehabilitation audit (2012, 2014, 2016, 2018 and 2020). The audit is designed to assess adherence to stroke clinical guidelines, and the questions included in each audit may change to align with updates to the clinical guidelines. In the present study, we only included questions from the audit that have been consistently collected in a standardized way over time. Efforts to improve data quality from these audits were in place in order to minimize bias associated with poor documentation, such as missing data. This included efforts to improve data quality by training data abstractors, having comprehensive data dictionaries, the use of in-built logic checks in data collection tools to minimize missing data, and having inter-rater reliability testing.^15^ Rehabilitation hospitals are generally independent to the acute settings, with few that have direct links with acute service providers.^16^ It is common practice for a full assessment of each patient admitted to the rehabilitation service be undertaken.^17^

To accurately ensure reliable estimates in adherence to acute processes, we limited our sample to hospitals that contributed ≥40 stroke admissions per acute audit cycle and ≥10 stroke admissions per rehabilitation audit cycle.^14^

The hospital-level mood management process that was assessed included the availability of clinical psychologist or neuropsychologist actively involved in the management of patients with stroke. However, this measure does not indicate the full-time equivalent (FTE) that is available.

The patient-level mood management processes that were assessed included (a) patients’ mood screened during admission, (b) patients with mood impairment if mood was assessed, (c) patients seen by a psychologist if mood was impaired and (d) mood management therapy used (e.g. anti-depressants, psychological therapy: variable available in rehabilitation audit only).

### Statistical analyses

All data were analyzed using Stata SE 17.0.^18^ A *p*-value <0.05 was considered statistically significant and all tests were two-tailed. Records with missing responses were considered incomplete and were therefore excluded from analyses using that variable. For the analyses of both acute and rehabilitation data, the following were undertaken:

Organizational survey: hospital-level analysis was performed using descriptive statistics to summarize the organizational survey data over audit cycles. Multilevel, multivariable logistic regression was used to assess temporal trends, with year included as an independent variable.

Clinical audit: patient-level analyses were performed, with chi-squared (categorical) and Wilcoxon rank-sum (continuous) tests used to compare patient characteristics over audit cycles. Multivariable, multilevel logistic regression was used to evaluate adherence over time, with the year of audit included as an independent variable, and level was defined as the hospital. These models were adjusted for all clinically relevant variables (e.g. age, sex, stroke type, stroke severity) and those identified in univariable tests with *p *< 0.1. We used the margins command to derive adjusted proportions and 95% confidence intervals for all multivariable models to allow for examination of the absolute, rather than relative, changes in adherence over time. Unadjusted and adjusted proportions were compared over time, using Cochrane Armitage tests for trends (*p*_trend_).

### Ethics

Hospital executives or the clinical lead of the stroke service of participating hospitals provided consent to participate in the audits. The Human Research Ethics Committee from Monash University (Project ID 35037) provided approval for this project.

## Results

Results are presented in two parts reflecting the acute and rehabilitation hospitals separately.

**Figure 1.**
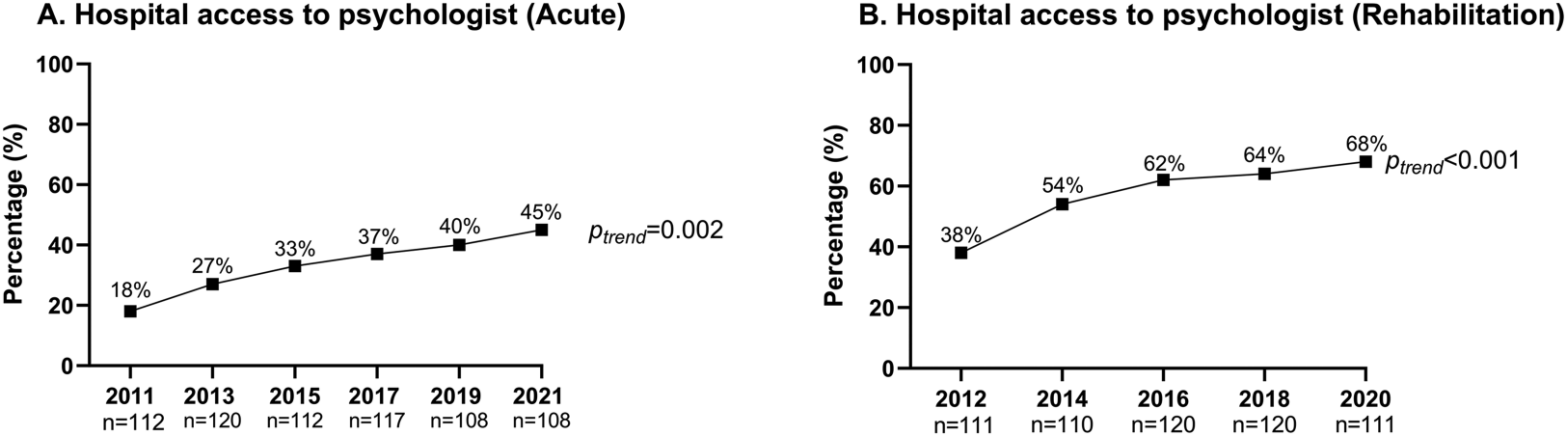
Proportion of hospitals with access to a psychologist for the management of patients with stroke in acute and rehabilitation hospitals across audit cycles.

Acute hospitals: between 2011 and 2021, a total of 142 hospitals participated in the acute services organizational survey. Overall, >95% of the participating acute hospitals in each cycle were public hospitals (Supplemental Table 1). As displayed in Figure 1(a), hospital access to a clinical psychologist or neuropsychologist in the management of patients with stroke increased from 18% in 2011 to 45% in 2021 (*p*_trend _= 0.002).

A total of 133 hospitals participated in the acute clinical audit between 2011 and 2021, with 22,937 cases included. Of the hospitals participating in the clinical audit, approximately 40% had between 75 and 199 stroke admissions per year and over 90% were public hospitals (Supplemental Table 2). An overview of the patient characteristics is provided in Table 1. Across the acute audit cycles, the median age ranged from 75 to 76 years, and under half of the patients in each audit was female (Table 1). There were significant differences in patient characteristics across the acute audit cycles. The characteristics from each audit cycle compared with the total cohort (all years) differed for the proportion of patients unable to walk on admission, speech and communication deficits, arm deficits, incontinence within 72 h of admission, and the median length of stay (Table 1).

**Table 1.** Aggregated and individual audit cycle patient characteristics for the acute clinical medical record audits.

Characteristics	Total cohort (2011–2021)	2011	2013	2015	2017	2019	2021
Number of hospitals	133	95	108	107	112	105	101
Number of cases	22,937	3415	3576	4012	4090	4026	3818
Variable	n (%)	n (%)	n (%)	n (%)	n (%)	n (%)	n (%)
Patient characteristics							
Age median (Q1, Q3)	76 (65, 84)	76* (65, 84)	76 (65, 84)	76 (65, 84)	75 (65, 84)	75 (65, 84)	75* (65, 83)
Female	10,246 (45)	1595* (47)	1623 (45)	1808 (45)	1841 (45)	1739 (43)	1640* (43)
Independence prior to stroke (mRS 0–2)	17,568 (79)	2273* (73)	2388* (73)	3210 (80)	3292* (80)	3311* (82)	3094* (81)
Cognitive deficit	7087 (35)	1262* (43)	1033 (36)	1223 (34)	1256 (33)	1180* (32)	1133* (31)
Interpreter required	1548 (7)	297* (9)	261 (7)	238 (6)	278 (7)	253 (6)	221* (6)
Patient history							
Prior stroke	4934 (24)	775* (28)	729 (25)	901* (26)	873 (23)	893 (23)	763* (21)
Prior ischemic heart disease	5447 (27)	904* (34)	899* (31)	991 (29)	944* (25)	961* (25)	748* (20)
Stroke type							
Ischemic	18,519 (81)	2560* (78)	2809* (80)	3162* (79)	3367 (82)	3376* (84)	3245* (85)
Haemorrhagic	2937 (13)	512* (16)	419 (12)	541 (13)	503 (12)	478 (12)	484 (13)
Undetermined/unknown	1301 (6)	221* (7)	290* (8)	309* (8)	220 (5)	172* (4)	89* (2)
Stroke severity indicators							
Unable to walk on admission	13,075 (59)	2162* (64)	2389* (69)	2161* (55)	2112* (54)	2206* (56)	2045* (55)
Speech/communication deficit on admission	12,385 (57)	2049* (64)	1730* (55)	2248* (59)	2243 (57)	2183 (56)	1932* (52)
Arm deficit on admission	13,512 (62)	2289* (70)	2050* (64)	2366 (62)	2356* (60)	2343* (60)	2108* (57)
Incontinence within 72 h	7578 (34)	1289* (40)	1277* (37)	1333 (34)	1350 (35)	1231* (31)	1098* (30)
Health system factors							
Managed in a stroke unit	15,428 (67)	2059* (60)	2165* (61)	2714 (68)	2893* (71)	2764 (69)	2833* (74)
Median length of stay, in days (Q1, Q3)	5 (3, 9)	6* (3, 11)	5 (2, 9)	5* (3, 10)	5 (3, 9)	5* (3, 9)	5* (2, 8)
Discharged to usual residence	10,843 (53)	1509 (51)	1570* (50)	1954* (55)	1886 (51)	2021* (55)	1903 (54)

Note: *p < .05, compared with total cohort (all years) using Chi-squared test or Wilcoxon rank-sum test. mRS: modified Rankin Scale; Q1: 25^th^ percentile; Q3: 75^th^ percentile.

**Figure 2.**
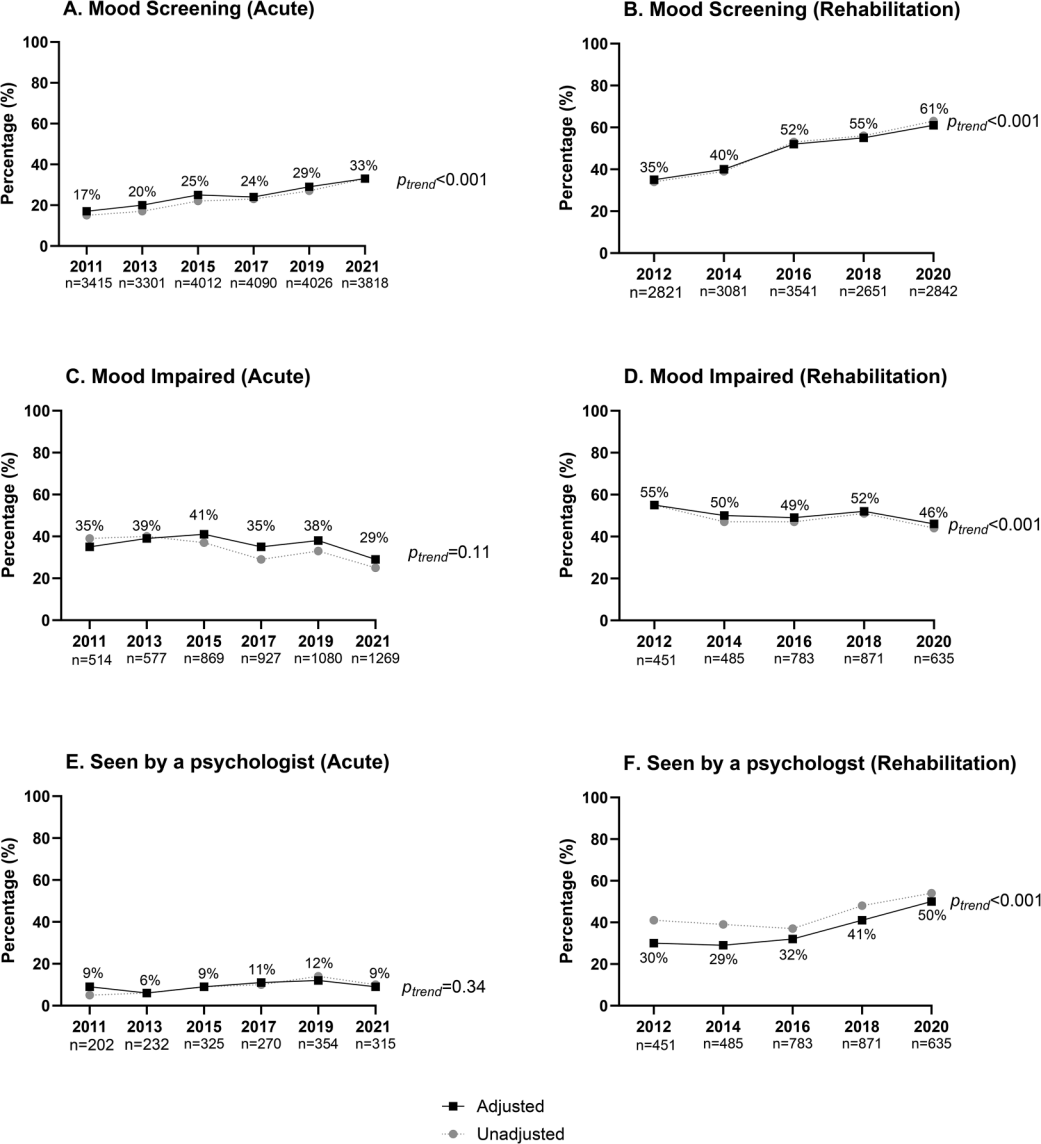
Proportion of episodes receiving recommended mood management processes in acute and rehabilitation hospitals across audit cycles.

Note: Adjusted line using covariates of age, sex, pre-morbid modified Rankin Scale (0–2), cognitive deficits (a and b only), interpreter required (a, b, c and f only), previous history of stroke (a and c), previous history of ischemic heart disease (a, c and e only), stroke type, ability to walk, speech impairment (a, c and e only), arm impairment, incontinence (a, c and e only), managed in a stroke unit (a, c and e only), >median length of stay and discharged to usual residence (a, c, d, e and f only), with level defined as hospital.

Changes over audit cycles for mood screening conducted in acute hospitals are shown in Figure 2(a). Within the acute audit, the adjusted proportion of patients who had their mood assessed during their admission increased from 17% in 2011 to 33% in 2021 (*p*_trend _< 0.001). Factors associated with receiving a mood screening included: younger age, independence prior to stroke, not requiring an interpreter, arm deficit, managed in a stroke unit, greater length of stay and not being discharged to usual residence (Table 2).

**Table 2.** Multivariable analysis for factors associated with adherence to mood management processes in acute and rehabilitation hospitals.

Number of cases receiving mood management process	Acute clinical audit		Rehabilitation clinical audit	
	Mood assessed	Seen by a psychologist if mood was impaired	Mood assessed	Seen by a psychologist if mood was impaired
	5214 (23%)	163 (10%)	7862 (49%)	1411 (44%)
	Odds-ratio (95% CI)	Odds-ratio (95% CI)	Odds-ratio (95% CI)	Odds-ratio (95% CI)
Patient characteristics				
Aged over 65	0.81* (0.73, 0.88)	0.55* (0.34, 0.90)	0.70* (0.64, 0.77)	0.64* (0.51, 0.81)
Male	1.05 (0.97, 1.14)	1.07 (0.69, 1.65)	0.99 (0.92, 1.06)	1.10 (0.90, 1.36)
Independence prior to stroke (mRS 0–2)	1.27* (1.13, 1.43)	1.35 (0.73, 2.50)	0.95 (0.82, 1.09)	0.83 (0.49, 1.42)
Cognitive deficit	1.05 (0.95, 1.15)	^a^	^c^	^c^
Interpreter required	0.70* (0.59, 0.84)	^a^	0.68* (0.59, 0.79)	0.64 (0.42, 0.97)
Patient history				
Prior stroke	1.07 (0.97, 1.19)	^a^	^c^	^c^
Prior ischemic heart disease	1.08 (0.98, 1.19)	0.91 (0.53, 1.56)	^c^	^c^
Stroke type				
Hemorrhagic^b^	0.89 (0.78, 1.03)	0.70 (0.34, 1.41)	1.00 (0.91, 1.11)	1.12 (0.86, 1.45)
Undetermined/unknown^b^	0.80* (0.64, 1.00)	0.35 (0.08, 1.62)	0.91 (0.78, 1.06)	1.77* (1.12, 2.78)
Stroke severity indicators				
Unable to walk on admission	1.01 (0.91, 1.11)	1.03 (0.61, 1.73)	1.10 (0.98, 1.22)	0.69* (0.49, 0.98)
Speech/communication deficit on admission	0.93 (0.85, 1.01)	0.74 (0.47, 1.17)	^c^	^c^
Arm deficit on admission	1.11* (1.01, 1.21)	0.94 (0.57, 1.56)	1.23* (1.13, 1.34)	1.14 (0.86, 1.49)
Incontinence within 72 h	0.96 (0.87, 1.07)	0.74 (0.44, 1.24)	^c^	^c^
Health system factors				
Managed in a stroke unit	1.55* (1.38, 1.75)	1.51 (0.73, 3.10)	^c^	^c^
Length of stay less than median (5 days in acute, 22 days in rehabilitation)	0.54* (0.49, 0.59)	0.12* (0.06, 0.24)	0.50* (0.47, 0.55)	0.47* (0.37, 0.60)
Discharged to usual residence	0.87* (0.79, 0.95)	0.73 (0.44, 1.21)	^a^	1.22 (0.98, 1.52)

Note: *p < .05. Model also adjusted for year of audit. mRS: modified Rankin Scale; CI: Confidence Intervals. ^a^ Variable not included in the multivariable analysis due to a *p *> 0.1 on univariate test. ^b^ Ischemic stroke type used as reference category. ^c^ Variable not included in the multivariable analysis due to variable not consistently reported in rehabilitation audit.

Of those who had their mood screened, the adjusted proportion of patients who were reported to have mood impairment decreased from 35% in 2011 to 29% in 2021, though this difference was not significant (*p*_trend _= 0.11; Figure 2(c)).

The adjusted percentage of patients seen by a psychologist if the patient's mood was impaired did not change over the audit cycles (2011—9%; 2021—9%, *p*_trend _= 0.34; Figure 2(e)). Factors associated with being seen by a psychologist if mood was impaired included: younger age and greater length of stay (Table 2).

Rehabilitation hospitals: between 2012 and 2020, a total of 130 hospitals participated in the rehabilitation hospitals organizational survey. Overall, >85% of the participating rehabilitation hospitals in each cycle were public hospitals (Supplemental Table 1). As displayed in Figure 1(b), hospital access to a clinical psychologist or neuropsychologist actively involved in the management of patients with stroke increased from 38% in 2012 to 68% in 2020 (*p*_trend _< 0.001).

A total of 127 hospitals participated in the rehabilitation clinical audit between 2012 and 2020, with 15,891 cases included. Of the hospitals participating in the clinical audit, approximately half had between 30 and 79 stroke admissions per year and approximately 90% were public (Supplemental Table 2). Across the rehabilitation audit cycles, median age ranged from 75 to 76 years, and under half of the patients in each audit were female (Table 3).

**Table 3.** Aggregated and individual audit cycle patient characteristics for the rehabilitation clinical medical record audits.

Characteristics	Total cohort (2012–2020)	2012	2014	2016	2018	2020
Number of hospitals	127	101	104	108	109	90
Number of cases	15,891	2821	3070	3507	3651	2842
Variable	n (%)	n (%)	n (%)	n (%)	n (%)	n (%)
Patient characteristics						
Age median (Q1, Q3)	76 (66, 84)	76 (66, 83)	76 (66, 84)	76 (66, 84)	76 (66, 83)	75* (65, 83)
Female	7148 (45%)	1288 (46%)	1420 (46%)	1548 (44%)	1656 (45%)	1236 (43%)
Independence prior to stroke (mRS 0–2)	14,277 (91%)	2569* (93%)	2770 (91%)	3102* (89%)	3300 (90%)	2536 (89%)
Interpreter required	1164 (7%)	284* (10%)	243* (8%)	204* (6%)	231 (6%)	202 (7%)
Stroke type						
Ischemic	5765 (58%)	2136 (76%)	2380* (78%)	2782* (79%)	2630* (72%)	2078* (73%)
Hemorrhagic	2782 (18%)	519 (18%)	532 (17%)	655 (19%)	605 (17%)	471 (17%)
Undetermined/unknown	1113 (7%)	166* (6%)	158* (5%)	70* (2%)	426* (12%)	293* (10%)
Stroke severity indicators						
Unable to walk on admission	12,476 (79%)	2352* (84%)	2564* (84%)	2611* (75%)	2785* (76%)	2163* (76%)
Arm deficit on admission	10,479 (71%)	1637 (69%)	1812 (69%)	2356* (69%)	2646* (73%)	2028 (73%)
Health system factors						
Median length of stay, in days (Q1, Q3)	22 (13, 39)	26* (14, 43)	22 (12, 39)	21* (12, 37)	22 (13, 38)	22 (13, 37)
Discharged to usual residence	10,378 (66%)	1934* (69%)	2100* (69%)	2267 (65%)	2252 (62%)	1825 (65%)

Note: mRS: modified Rankin Scale; Q1: Quartile 1; Q3: Quartile 3. *p < 0.05, compared with reference year (2012–2020) using Chi-squared test or Wilcoxon rank-sum test.

Within the rehabilitation audit, the adjusted number of patients who had their mood assessed during their admission (Figure 2(b)) increased from 35% in 2012 to 61% in 2020 (*p*_trend _< 0.001). Factors associated with receiving a mood screening included: younger age, not requiring an interpreter, arm deficit and greater length of stay (Table 2).

The adjusted proportion of patients who were reported to have mood impairment after a mood screening (Figure 2(d)) decreased from 55% in 2012 to 46% in 2020 (*p*_trend _< 0.001).

An increase in the adjusted proportion of patients seen by a psychologist if the patient's mood was impaired was observed (Figure 2(f)), from 30% in 2012 to 50% in 2020 (*p*_trend _< 0.001). Factors associated with being seen by a psychologist if mood was impaired included: younger age, not requiring an interpreter, able to walk and greater length of stay.

**Figure 3.**
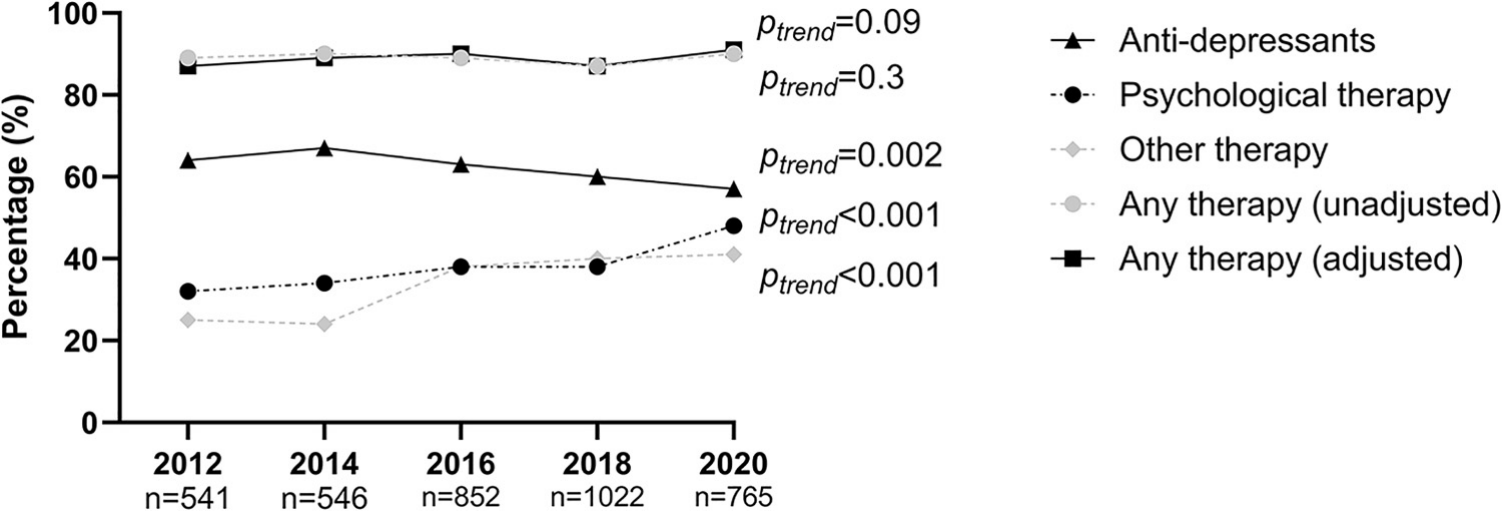
Mood therapy used among patients with stroke if mood was impaired, in rehabilitation hospitals across audit cycles.

Note: Adjusted using year as an independent variable, as well as covariates of age, sex, pre-morbid modified Rankin Scale (0–2), inability to walk on admission, arm deficit, >median length of stay and discharged to usual residence, with level defined as hospital.

The adjusted proportion of therapies used if mood was impaired are shown in Figure 3. The adjusted proportion of any therapy being provided as a result of mood impairment did not significantly change over the audit cycles (87% in 2012; 91% in 2020, *p*_trend _= 0.09). Anti-depressants used as therapy if mood was impaired decreased from 64% in 2012 to 57% in 2020 (*p*_trend _= 0.002). Psychological therapy used if mood was impaired increased from 32% in 2012 to 48% in 2020 (*p*_trend _< 0.001). Other therapies used if mood was impaired increased from 25% in 2012 to 41% in 2020 (*p*_trend _< 0.001). Details of these other therapies were not recorded.

## Discussion

Our study provides evidence that improvements have been made in mood screening and management of people with stroke admitted to Australian acute and rehabilitation hospitals over a 10-year period. Results from acute and rehabilitation hospitals were analyzed independently to determine whether changes were limited to a particular healthcare setting.

Within acute hospitals, there was an increase in the proportion of stroke services that had access to a psychologist, and an increase in the proportion of patients with stroke who had their mood screened. However, these proportions in 2021 remained low (45% and 33%, respectively). These results align with previous research conducted in the UK, where access to psychological services,^19^ and compliance with post-stroke mood screening within inpatient services was poor.^20,21^ We found an increase in the total number of mood screenings undertaken each year and people with a mood impairment, despite the proportion of patients with mood impairment not significantly changing over the audit cycles. Despite an increase in hospitals with psychologists involved in managing stroke patients, the percentage of stroke patients with mood disorders who received psychological care remained unchanged between 2011 and 2021. Notably, other multidisciplinary team members, such as social workers, may have been utilized instead,^22^ which the data from these audits do not detail.

Australian rehabilitation hospitals showed greater overall improvements than acute hospitals. Psychologists became more available for stroke patients, and more patients had their mood screened. However, only half of patients with mood impairment were seen by a psychologist in 2020. Similar results have been observed in the UK, where there was a higher level of psychologist input in the rehabilitation inpatient setting compared with the acute patient setting.^23^ Nevertheless, accessing post-acute psychological support within the UK also remains a challenge.^23^

No increasing trends in the overall provision of mood therapies for those with a mood impairment were found. However, antidepressant prescription decreased while psychological therapy increased between 2012 and 2020. Antidepressants, have a strong evidence-base for their usage post-stroke and are currently the most popular form of treatment for post-stroke depression.^13,24^ The evidence base for psychological therapies has grown over the past decade,^24,25^ which may explain our observations. To the best of our knowledge, this is the first study to observe a change over time in therapies provided for managing mental health impairments post-stroke. The use of other therapies also increased between 2012 and 2020. Future audits should attempt to capture what alternative therapies are being provided for mood impairment after stroke.

Factors associated with receiving mood management processes within both acute and rehabilitation hospitals included: age under 65 years, not requiring an interpreter for communication, and a longer length of stay. Younger age has previously been shown to influence the management of care received post-stroke, with younger patients more likely to receive evidence-based stroke care and information about community reintegration.^26^ Authors of previous studies have also acknowledged disparities in care provision for those requiring an interpreter while in hospital.^27,28^ Therapists have reported challenges assessing mood without subtleties of language,^29^ and cultural stigmas to mental health may create additional barrier for assessing mood.^30^ Many hospitals attempt to reduce length of stay to reduce hospital costs and improve capacity.^31^ This may result in patients being discharged too early for a mood assessment to be conducted.^32^ Other factors related to receiving some mood management processes included stroke type and severity indicators, and management in a stroke unit, which are known to influence receipt of care post-stroke.^33,34^

Our study highlights the need for strategies to improve use of mood screening. However, conducting mood screens alone is insufficient to achieve optimal disease management. A pathway for psychological care for those identified by the screening tools also needs to be in place,^35^ including using appropriate screening tools for people with aphasia or cognitive difficulties.^36^ Screening tools with an incorporated pathway for assessment and management of anxiety and depression post-stroke have been developed.^36–38^ McLean et al.^22^ adapted one such pathway for their hospital. The authors showed that the pathway improved rates of screening, clinical interviews and interventions for post-stroke mood impairments for patients who were admitted to the rehabilitation hospital. The three-step process is now recommended in the UK with criteria for use established for the hospital system. Further research is needed to increase the evidence to support the implementation of similar pathways for Australia.

Several limitations of our study exist. Participation in the Audit Program is voluntary and hospitals participating within each audit may differ. To maintain homogeneity, only acute hospitals with over 40 annual stroke admissions per year, and rehabilitation hospitals with greater than 10 annual stroke admission per year, were included.^39^ The de-identified and cross-sectional nature of these data prevented us from linking person-level information over multiple audits. Given this study was focused on evaluating care quality from a system-level perspective, this limitation is unlikely to alter the study conclusions.^17^ Data that were not available for all audit cycles could not be used in the analysis. These included cognitive deficits, incontinence, speech impairment (not recorded within the rehabilitation audit) and information on staffing levels in each facility. Further, information on the diagnosis of, and treatment for, a mental health impairment prior to the audited admission was unavailable; this is known to influence utilization of mental health services.^40^ Caution must be taken with generalizability of the current study to other developed countries, as each health system functions in its own local context.

Although improvements in conducting mood screening and access to psychologists’ were made over 10 years, mood management processes still remain poor within Australian acute and rehabilitation stroke hospitals. Many patients are still being discharged from acute and inpatient rehabilitation settings without receiving a mood screen. In particular, access to a psychologist for those with a mood impairment within acute settings has not improved and warrants further investigation. Additionally, those aged over 65, requiring an interpreter, and with shorter hospital lengths of stay were found to have less access, illustrating equality issues.

Clinical message

- Mood management within Australian stroke hospitals has improved, but still remains poor.
- There needs to be a greater focus on mood management for patients aged 65+, who require an interpreter, or who have a shorter length of stay.
- Establishment of psychological care pathways may assist in improving equity to mood screening.

## Supplemental Material

sj-docx-1-cre-10.1177_02692155241232990 - Supplemental material for Access to inpatient mood management services after stroke in Australian acute and rehabilitation hospitals Supplemental material, sj-docx-1-cre-10.1177_02692155241232990 for Access to inpatient mood management services after stroke in Australian acute and rehabilitation hospitals by Shaun L Hancock, Tara Purvis, Tharshanah Thayabaranathan, Rene Stolwyk, Jan Cameron, Lachlan L Dalli, Megan Reyneke, Monique F Kilkenny, Kelvin Hill and Dominique A Cadilhac in Clinical Rehabilitation

sj-docx-2-cre-10.1177_02692155241232990 - Supplemental material for Access to inpatient mood management services after stroke in Australian acute and rehabilitation hospitals Supplemental material, sj-docx-2-cre-10.1177_02692155241232990 for Access to inpatient mood management services after stroke in Australian acute and rehabilitation hospitals by Shaun L Hancock, Tara Purvis, Tharshanah Thayabaranathan, Rene Stolwyk, Jan Cameron, Lachlan L Dalli, Megan Reyneke, Monique F Kilkenny, Kelvin Hill and Dominique A Cadilhac in Clinical Rehabilitation
