# Supplementary material for: Access to inpatient mood management services after stroke in Australian acute and rehabilitation hospitals
Source: Clin Rehabil. 2024 Feb 22;38(6):811–23. doi: 10.1177/02692155241232990 (PMC11059847; doi:10.1177/02692155241232990)
Supplement: sj-docx-1-cre-10.1177_02692155241232990 - Supplemental material for Access to inpatient mood management services after stroke in Australian acute and rehabilitation hospitals [file sj-docx-1-cre-10.1177_02692155241232990.docx]

Supplementary Table 1. Organizational survey details for participating hospitals

| Acute Organizational Survey | | | | | | |
| --- | --- | --- | --- | --- | --- | --- |
|  | 2011  N = 112 | 2013  N = 120 | 2015  N = 112 | 2017  N = 117 | 2019  N = 108 | 2021  N = 108 |
| Annual stroke admissions | | |  |  |  |  |
| <75 | 21 (19%) | 29 (24%) | 20 (18%) | 22 (19%) | 13 (12%) | 14 (13%) |
| 75-199 | 44 (39%) | 41 (34%) | 38 (34%) | 35 (30%) | 32 (30%) | 29 (27%) |
| 200-349 | 24 (21%) | 23 (19%) | 29 (26%) | 33 (28%) | 28 (26%) | 27 (25%) |
| 350-499 | 13 (12%) | 13 (11%) | 8 (7%) | 5 (4%) | 11 (10%) | 18 (17%) |
| ≥500 | 10 (9%) | 14 (12%) | 17 (15%) | 22 (19%) | 24 (22%) | 20 (19%) |
| Public setting | 109 (97%) | 115 (96%) | 107 (96%) | 112 (96%) | 104 (96%) | 104 (96%) |
| Rehabilitation Organizational Survey | | | | | | |
|  | 2012  N = 111 | 2014  N = 110 | 2016  N = 120 | 2018  N = 120 | 2020  N = 111 |  |
| Annual stroke admissions | |  |  |  |  |  |
| <30 | 25 (23%) | 16 (15%) | 21 (18%) | 19 (16%) | 23 (21%) |  |
| 30-79 | 61 (55%) | 57 (52%) | 58 (48%) | 62 (52%) | 54 (48%) |  |
| ≥80 | 25 (23%) | 37 (34%) | 41 (34%) | 39 (33%) | 35 (32%) |  |
| Public setting | 98 (88%) | 95 (86%) | 102 (85%) | 103 (86%) | 96 (86%) |  |
