# Supplementary material for: Access to inpatient mood management services after stroke in Australian acute and rehabilitation hospitals
Source: Clin Rehabil. 2024 Feb 22;38(6):811–23. doi: 10.1177/02692155241232990 (PMC11059847; doi:10.1177/02692155241232990)
Supplement: sj-docx-2-cre-10.1177_02692155241232990 - Supplemental material for Access to inpatient mood management services after stroke in Australian acute and rehabilitation hospitals [file sj-docx-2-cre-10.1177_02692155241232990.docx]

Supplementary Table 2. Site participation for the Acute and Rehabilitation clinical medical record audits

| Acute Audit | 2011 | | 2013 | | 2015 | | 2017 | | 2019 | | 2021 | |
| --- | --- | --- | --- | --- | --- | --- | --- | --- | --- | --- | --- | --- |
| Clinical Audit Participation | Hospitals | Cases | Hospitals | Cases | Hospitals | Cases | Hospitals | Cases | Hospitals | Cases | Hospitals | Cases |
| N | 95 | 3415 | 108 | 3576 | 107 | 4012 | 112 | 4090 | 105 | 4026 | 101 | 3818 |
| Annual stroke admissions |  |  |  |  |  |  |  |  |  |  |  |  |
| <75 | 13 (14%) | 258 (8%) | 21 (19%) | 415 (12%) | 18 (17%) | 474 (12%) | 18 (16%) | 396 (10%) | 11 (10%) | 296 (7%) | 9 (9%) | 192 (5%) |
| 75-199 | 39 (41%) | 1,299 (38%) | 38 (35%) | 1331 (37%) | 37 (35%) | 1301 (32%) | 35 (31%) | 1,283 (31%) | 31 (30%) | 1,104 (27%) | 28 (28%) | 1,013 (27%) |
| 200-349 | 21 (22%) | 779 (23%) | 23 (21%) | 811 (23%) | 27 (25%) | 1,057 (26%) | 32 (29%) | 1,247 (30%) | 28 (27%) | 1106 (27%) | 26 (26%) | 1,012 (27%) |
| 350-499 | 12 (13%) | 547 (16%) | 12 (11%) | 443 (12%) | 8 (7%) | 332 (8%) | 5 (4%) | 201 (5%) | 11 (10%) | 464 (12%) | 18 (18%) | 750 (20%) |
| ≥500 | 10 (11%) | 532 (16%) | 14 (13%) | 576 (16%) | 17 (16%) | 848 (21%) | 22 (20%) | 963 (24%) | 24 (23%) | 1,056 (26%) | 20 (20%) | 851 (22%) |
| Public setting | 93 (98%) | 3,346 (98%) | 103 (95%) | 3,405 (95%) | 102 (95%) | 3,811 (95%) | 108 (96%) | 3,978 (97%) | 101 (96%) | 3,890 (97%) | 99 (98%) | 3,736 (98%) |
| Rehabilitation Audit | 2012 | | 2014 | | 2016 | | 2018 | | 2020 | |  |  |
| Clinical Audit Participation | Hospitals | Cases | Hospitals | Cases | Hospitals | Cases | Hospitals | Cases | Hospitals | Cases |  |  |
| N | 101 | 2821 | 102 | 3070 | 107 | 3507 | 109 | 3651 | 90 | 2842 |  |  |
| Annual stroke admissions |  |  |  |  |  |  |  |  |  |  |  |  |
| <30 | 22 (22%) | 351 (12%) | 15 (15%) | 237 (8%) | 17 (16%) | 331 (13%) | 17 (16%) | 327 (9%) | 14 (16%) | 243 (9%) |  |  |
| 30-79 | 55 (54%) | 1,658 (59%) | 53 (52%) | 1,664 (54%) | 54 (50%) | 1,860 (74%) | 56 (51%) | 1,930 (53%) | 48 (53%) | 1,528 (54%) |  |  |
| ≥80 | 24 (24%) | 812 (29%) | 34 (33%) | 1,169 (38%) | 36 (34%) | 1,316 (52%) | 36 (33%) | 1,394 (38%) | 28 (31%) | 1,071 (38%) |  |  |
| Public setting | 89 (88%) | 2,542 (90%) | 90 (88%) | 2,721 (89%) | 92 (86%) | 3,062 (122%) | 95 (87%) | 3,246 (89%) | 81 (90%) | 2,541 (89%) |  |  |
